# Assessment of Microbial and Heavy Metal Contamination of Natural Sheep Casings from Different Geographic Regions

**DOI:** 10.3390/foods14091520

**Published:** 2025-04-26

**Authors:** Beata Wysok, Adam Dymkowski, Marta Sołtysiuk, Aleksandra Kobuszewska

**Affiliations:** Department of Veterinary Public Health, Faculty of Veterinary Medicine, University of Warmia and Mazury in Olsztyn, Oczapowskiego 2, 10-917 Olsztyn, Poland; 152342@student.uwm.edu.pl (A.D.); marta.soltysiuk@uwm.edu.pl (M.S.); aleksandra.kobuszewska@uwm.edu.pl (A.K.)

**Keywords:** natural casings, heavy metals, microbial contamination of foodstuffs

## Abstract

Natural casings are integral components in the production of various meat products, including sausages, and their quality and safety have to be controlled to eliminate any risks to consumers’ health. A total of 35 samples of salted natural sheep casings from Turkey, Iran, China, Mongolia, Pakistan, New Zealand, the United Kingdom, and Belgium were tested for microbial contamination and the concentrations of potentially toxic heavy metals. The mean log values of microbial counts were determined at 3.45 ± 0.44 log CFU/g for aerobic mesophilic bacteria, 0.5 ± 0.43 log CFU/g for anaerobic sulfide-reducing bacteria, and 1.24 ± 0.63 log CFU/g for coagulase-positive staphylococci. Typical or suspected colonies of *Salmonella* spp., *E. coli*, and *Listeria* spp. were not identified on selective and differential agar. The examined casings were contaminated mainly with lead (0.077 ± 0.045 mg/kg), followed by arsenic (0.036 ± 0.029 mg/kg) and cadmium (0.009 ± 0.008 mg/kg). The concentrations of mercury in all samples were below the limit of quantification. The study demonstrated that the quality and safety of natural casings were not affected by their region of origin and that microbial contamination was not correlated with heavy metal concentrations.

## 1. Introduction

In recent years, consumer interest in processed meat products such as sausages and other processed foods, as well as ready-made food products, has increased around the world. Casings are an integral part of sausages; they ensure the shape, size, and integrity of sausages, and their functional role begins during stuffing and ends at the table [1]. Both natural and artificial casings display these properties. Natural casings are made from the submucosa of a farm animal’s intestines. In turn, artificial casings are manufactured from biopolymers such as cellulose and collagen. Artificial casings separate meat products from the surrounding environment and transform them into integral units. In turn, natural casings allow water vapor and gases to permeate the product and enhance the traditional taste of sausages [2]. Most natural casings are made from sheep and hog intestines. Sheep casings are regarded as most tender and delicate, but they are also sufficiently tough, less susceptible to chewing, and offer a superior flavor [3]. Natural casings play an important role in the production of various foodstuffs, and their microbiological status is crucial to ensuring food safety. The processing of natural casings includes the salting stage to obtain products of the highest quality and to eliminate microbiological risks for consumers. Salt is the main preservative that eliminates vegetative bacteria by removing water from bacterial cells and causing cell death by osmotic shock [4]. Based on the European Union’s Community Guide to Good Practice for Hygiene and HACCP principles for the production of natural sausage casings (accessed in December 2024), natural sausage casings should be salted in dry salt (NaCl) or brine (saturated salt solution) for at least 30 days to obtain the final product. Despite the fact that salting is a food preservation method that relies on osmotic dehydration, it may not inhibit the growth of some pathogenic bacteria. Bacteria that are resistant to multiple environmental stressors, are able to survive in food processing environments, and grow in high-salt foods include *Listeria* spp., *Salmonella* spp., *Escherichia coli*, *Staphylococci*, and sulfate-reducing bacteria of the genus *Clostridium*. It is generally assumed that *Clostridium* can be present in soil or feces; contamination with *Salmonella, E. coli* or *Listeria* occurs through feces, the environment, and humans, whereas contamination with *Staphylococcus aureus* takes place mainly through contact with human skin carrying these pathogens. Chemical factors, mainly trace elements, also pose potential risks during the production of natural casings. Natural casings are generally processed with the use of food-grade salt, which should not contain heavy metals above the maximum permissible limits. Incidences of heavy metal contamination of salt have been reported worldwide in recent years [5]. It should be noted that the geographic origin of salt, including salt mining sites and processing techniques, significantly contributes to the content of toxic mineral elements. Lead (Pb), cadmium (Cd), mercury (Hg), and arsenic (As) are heavy metals that pose the greatest risk of food contamination [6]. Heavy metals are non-biodegradable and persistent contaminants that accumulate in the major organs of the human body, including the kidneys, bones, and liver, and are associated with many serious health disorders [7].

Salted natural casings are supplied with a trade identification document, which is the exporter’s declaration of the origin of the product. This document contains basic data regarding the name and address of the manufacturer, the origin of the product, the batch identification, date of production, date of shipment, and place of destination. There is no information about processing and preparation of the final product. The aim of this study was to compare microbial contamination levels and the concentrations of potentially toxic heavy metals in natural sheep casings derived from suppliers from different countries to compare hygienic standards of the final product ready to process.

## 2. Materials and Methods

### 2.1. Samples

Salted natural sheep casings (20–22 mm in diameter), constituting the submucosal layer of the small intestine, produced in Turkey, Iran, China, Mongolia, Pakistan, New Zealand, the United Kingdom, and Belgium were tested. All materials were obtained from a Polish distributor of natural casings from various suppliers. The casings were imported from the country of origin in closed containers with an estimated capacity of 160–200 L. The sampling process was designed to take into account the quality of casings from different deliveries representing various batches. For this purpose, the samples were collected randomly from 20% of containers from each delivery. From each selected container, three subsamples from the bottom, middle, and top parts were collected, and subsequently, all individual samples were pooled. Samples of salted casings were stored at 4 °C for analysis.

### 2.2. Physicochemical Properties

Water activity (a_w_) was measured using an AwTherm–Water Activity meter (Rotronic, Bassersdorf, Switzerland). The samples were prepared by pulverizing 20 g of the pooled samples of natural casings using a hand blender.

For pH analysis, 10 g of each sample was homogenized with 90 mL distilled water for 1 min and measured using a digital pH meter AD1020 Professional Multi-Parameter pH-ORP-ISE-TEMP Bench Meter (Adwa Instruments, Szeged, Hungary). For each sample tested, the *a*_w_ and pH was determined in triplicate the averaged values were calculated.

### 2.3. Microbiological Analysis

For microbial analysis, 200 g of pooled samples was washed and desalted for 2 h in running high-purity deionized water (resistivity of 18.2 MWcm) supplied by the Ultrapure Millipore Direct-Q-R 3UV system (Merc Millipore, Darmstadt, Germany) before analysis. The samples were analyzed immediately after desalting. The results representing each batch from various suppliers of different countries of origin are given in Table 1.

The enumeration of aerobic bacteria and coagulase-positive staphylococci, as well as the presence of *Salmonella* spp. and *Listeria monocytogenes*, were determined according to the Bacteriological Analytical Manual (BAM) of the Food and Drug Administration (FDA). Anaerobic sulfide-reducing bacteria and *E. coli* were enumerated according to the ISO standards given below.

#### 2.3.1. Total Counts of Aerobic Mesophilic Bacteria

The total counts of aerobic mesophilic bacteria were determined by plating serial dilutions on Plate Count Agar (PCA) (Oxoid, Basingstoke, UK). For the initial dilution, 25 g of desalted natural casings was transferred to sterile bags containing 225 mL of Phosphate Buffered Saline (PBS) (Oxoid, Basingstoke, UK) and homogenized in a stomacher. Subsequently, 1 mL of each dilution was spread in separate, duplicate Petri plates, and 12–15 mL of PCA (cooled to 45 ± 1 °C) was poured over the sample on each plate. Petri plates were rotated to combine the dilution with the agar and were left to solidify. Bacteria were enumerated after 48 h of incubation at 37 °C, and the results were expressed in log_10_ CFU/g [8].

#### 2.3.2. Enumeration of *Escherichia coli*

The enumeration of *E. coli* was carried out according to ISO 16649-2:2001 [9]. Twenty-five grams of the sample was homogenized with 225 mL of Maximum Recovery Diluent (MRD). Serial dilutions were prepared from the original homogenate in MRD. Then, 1 mL of each dilution was spread into duplicate Petri plates, and 12–15 mL of Tryptone Bile X-Glucuronide agar (TBX) (Oxoid, Basingstoke, UK) was poured over the sample. Next, Petri dishes were rotated to distribute bacteria evenly and were left to solidify. Bacteria were enumerated after incubation at 44 °C for 24 h.

#### 2.3.3. Determination of *Salmonella* spp.

The presence of *Salmonella* spp. was determined in 25 g samples suspended in 225 mL of Buffered Peptone Water (BPW) (Oxoid, Basingstoke, UK). After incubation at 37 °C for 24 h, 1 mL of the obtained culture was transferred to 9 mL of Muller Kauffmann Tetrathionate Novobiocin Broth medium (MKTTn) (Oxoid, Basingstoke, UK) and 0.1 mL of Rappaport-Vassiliadis Soya Peptone Broth medium (RVS) (Oxoid, Basingstoke, UK). After incubation at 37 °C for 24 h and 42 °C for 24 h, respectively, the suspensions were transferred to selective agar plates, including Xylose Lysine Deoxycholate agar (XLD) (Oxoid, Basingstoke, UK) and Hektoen Enteric agar (HE) (Oxoid, Basingstoke, UK) [10]. After incubation, the colonies that emerged were analyzed as described below.

#### 2.3.4. Determination of *Listeria* spp.

The presence of *Listeria* spp. was determined in 25 g samples suspended in 225 mL of Buffered Listeria Enrichment Broth (BLEB) (Oxoid, Basingstoke, UK) enriched with the recommended supplements. After incubation at 30 °C for 24–48 h, 100 µL of the enriched cultures were streaked onto Oxford agar and PALCAM agar (Oxoid, Basingstoke, UK) [11]. The inoculated plates were incubated again, and the colonies that emerged were analyzed as described below.

#### 2.3.5. Determination of Coagulase-Positive Staphylococci

Coagulase-positive staphylococci were enumerated by plating serial dilutions on Baird-Parker agar (Oxoid, Basingstoke, UK). The initial dilution was prepared according to the same protocol that was applied to determine the total counts of aerobic mesophilic bacteria. Then, 100 µL of the suspension was streaked onto Baird-Parker agar; the plates were incubated at 37 °C for 24–48 h, and the colonies that emerged were analyzed as described below [12].

#### 2.3.6. Determination of Anaerobic Sulfide-Reducing Bacteria

Anaerobic sulfide-reducing bacteria were determined according to ISO 15213-1:2023 [13], with some modifications. Duplicate samples of 10 g each were suspended in 90 mL of Buffered Peptone Water (Oxoid, Basingstoke, UK). Before incubation, one of the samples was heated at 80 °C for 10 min to remove vegetative forms of spore-forming bacteria and/or non-spore-forming bacteria. A 10-fold dilution was prepared, and the suspension was spread onto Iron Sulfite Agar (ISA) (Oxoid, Basingstoke, UK). Petri plates were incubated at 37 °C for 24–48 h under anaerobic conditions, and the colonies that emerged were analyzed as described below.

### 2.4. Bacterial Identification

At least two colonies grown on selective media were picked up to identify bacteria to the species level. Bacteria were identified by matrix-assisted laser desorption/ionization–time-of-flight mass spectrometry (MALDI-TOF MS; Bruker, Karlsruhe, Germany). The picked-up colonies were subcultured on Columbia agar containing 5% sheep blood (Merc, Darmstadt, Germany) at 37 °C for 24 to 48 h in a suitable incubation atmosphere. Freshly grown overnight colonies were spotted onto MALDI-TOF target plates and overlaid with 1 µL of 70% formic acid (Merck, Darmstadt, Germany). Each spot was allowed to dry and was then overlaid with 1 µL of the matrix (α-cyano-4-hydroxycinnamic acid) (Merck, Darmstadt, Germany). Mass spectra were acquired in linear positive mode with a mass-to-charge ratio (m/z) of 2000 to 20,000. The spectra were evaluated in the MALDI BioTyper system with BioTyper v. 3.1 software (Bruker Daltonics GmbH, Bremen, Germany); scores of ≥2.0 were accepted for reliable identification at the species level, scores of ≥1.7 and ≤2.0 were accepted for identification at the genus level, and scores of <1.7 were considered unreliable.

### 2.5. Determination of Trace Elements

The concentrations of arsenic, cadmium, mercury, and lead in salted sheep casings were determined by inductively coupled plasma mass spectrometry (ICP-MS). Homogenized samples of 30 mg each were placed in quartz digestion vessels containing 8 mL of nitric acid (65%) (Merck, Darmstadt, Germany) and 1 mL of hydrogen peroxide (30%) (Merck, Darmstadt, Germany) and were digested in a closed microwave digestion system (ETHOS One, Milestone, Shelton, CT, USA) at 180 °C (1000 W; ramp time—25 min; hold time—15 min). The digested samples were cooled, 0.5 mL of hydrochloric acid was added, and the samples were diluted to a final volume of 25 mL using ultrapure water (Merck, Darmstadt, Germany). Lead, mercury, arsenic, and cadmium were quantified in the Agilent 7850 ICP-MS system (Agilent Technologies, Santa Clara, CA, USA) under the following conditions: RF power—1600 W; carrier gas flow rate—0.8 L/min; dilution gas flow rate—0.15 L/min; helium cell gas flow rate—4.3 mL/min; energy discrimination—5.0 V, with calibration standards matched from 0 to 100 µg/L for all elements. The limits of quantification (LOQs) were 0.001 mg/kg for Cd and Hg, and 0.01 mg/kg for As and Pb. The concentrations of trace elements were expressed in mg/kg on a dry matter (DM) basis.

### 2.6. Statistical Analysis

Significant differences in bacterial counts between samples were determined with a 2 × 2 contingency table. The mean concentrations of heavy metals in samples from different geographic regions were compared using Tukey’s post hoc test (Statistica, Krakow, Poland). The level of significance was set at *p* < 0.05.

## 3. Results

### 3.1. Physicochemical Analysis

The water activity (a_w_) of the sheep natural casings was at the level of 0.742 in samples from Turkey, 0.784 from Iran, 0.733 from China, 0.810 from Mongolia, 0.754 from Pakistan, 0.743 from New Zealand, 0.774 from the United Kingdom, and 0.789 from Belgium.

As for pH, the noted values were 7.6 in samples from Turkey, 7.3 from Iran, 7.7 from China, 7.4 from Mongolia, 7.6 from Pakistan, 7.8 from New Zealand, 7.7 from the United Kingdom, and 7.3 from Belgium.

### 3.2. Microbial Contamination

The mean log value of the total counts of aerobic mesophilic bacteria in the 35 tested samples was 3.45 ± 0.44 log CFU/g. The highest values were noted in Chinese casings (4.08 ± 3.16 log CFU/g), and the lowest values were noted in casings produced in the UK (2.91 ± 2.23 log CFU/g), but the observed differences were not significant (*p* = 0.291371) (Table 1). The spores of anaerobic sulfide-reducing bacteria (SRB) were present in 20% of the samples with a mean count of 0.5 ± 0.43 log CFU/g. Among samples positive for anaerobic SRB, microbial counts ranged from 0.6 log CFU/g in casings from Pakistan to 0.97 ± 0.17 log CFU/g in casings from Iran (*p* = 0.010133). Coagulase-positive staphylococci (CoPS) were detected in 71.4% of the samples with a mean count of 1.24 ± 0.63 log CFU/g, with the lowest values in Belgian casings (1.02 log CFU/g) and the highest values in samples from Pakistan (1.93 log CFU/g) (*p* = 0.018152). Typical or suspected colonies of *Salmonella* spp. and *Listeria* spp. were not identified on selective and differential agar. However, individual colonies that emerged on selective media were identified to the species level by mass spectrometry. These colonies were identified as *Halomonas elongata* (three samples), *Halomonas eurihalina* (one sample), *Pseudomonas stutzeri* (two samples), and *Pseudomonas oryzihabitans* (one sample).

### 3.3. Heavy Metal Concentrations

Lead was the heavy metal with the highest mean concentration (0.077 ± 0.045 mg/kg), and its content ranged from 0.064 ± 0.048 mg/kg in samples from New Zealand to 0.097 ± 0.052 mg/kg in samples from Pakistan (*p* > 0.05). In all samples, the observed values were above the limit of quantification (LOQ). The mean levels of arsenic and cadmium were 0.036 ± 0.029 mg/kg and 0.009 ± 0.008 mg/kg, respectively. Arsenic and cadmium concentrations were below the LOQ in 11.4% and 22.9% of the tested samples, respectively. The highest observed values for arsenic were noted at the level of 0.13 mg/kg in a sample from Iran and for cadmium at the level of 0.026 mg/kg in a sample from Pakistan. Mercury concentrations were below the LOQ in all samples (Table 2).

## 4. Discussion

Natural casings are a critical component of traditional and popular sausages, but they are produced from the intestines of slaughtered livestock that are naturally contaminated with microorganisms, which could affect their quality and safety. The gut microbiome of ruminants consists of bacteria, archaea, fungi, and protozoa that form a unique ecosystem [14]. These abundant and diverse microbial populations consist of both saprophytic and pathogenic bacteria. In the production process of natural casings, the intestines are washed, and most of their inner and outer content is removed, which significantly decreases the bacterial load. Salting is the last step of the production process, and it plays a critical role in food preservation by lowering water activity (a_w_) and eliminating undesirable microorganisms [15]. Generally, it is assumed that natural casings, due to their intestinal source, are by their nature matrices rich in bacteria, with total loads that can vary between 10^4^ and 10^7^ CFU/g [16]. In this study, the final product, i.e., salted natural casings, was characterized by the total bacterial count at the mean level of 3.45 log CFU/g. However, the observed values differed between casings produced in various geographic regions, and the noted differences were statistically significant. The recommended values of fully acceptable salted natural casings as incoming products at meat processing establishments for total aerobic bacterial given in the European Union’s Community Guide to Good Practice for Hygiene and HACCP principles for the production of natural sausage casings (accessed on December 2024) were estimated at the level of 1.0 × 10^5^ CFU/g, which indicates that the bacterial contamination of the examined casings in the current study did not exceed this level in any sample. In a study by Chawla et al. [17], total viable counts (TVC) in fresh natural lamb casings were 5.5–5.9 log CFU/g and decreased to 4.7–5.3 log CFU/g after 30 days of storage in 10% (*w*/*w*) sodium chloride (food-grade table salt) and to 3.79–4.04 log CFU/g after 90 days of storage. According to Wijnker et al. [18], this reduction is not only the result of applying sodium chloride but is an effect of a number of variables. McMeekin et al. [19] revealed that the combined effects of water activity, temperature, and pH lead to a greater magnitude of microbial growth rate than the sum of constraints applied individually. Most bacteria require a_w_ > 0.90 for minimal growth; however, some halophilic or halotolerant bacteria are able to survive at high salt concentrations [20]. Preservation of natural casings in dry salt or saturated brine for at least 30 days allows an a_w_ of 0.75–0.80 to be achieved to effectively eliminate bacteria and can therefore be considered as a protective measure for the international trade in natural casings [21]. Moreover, Wijnker et al. [18] noted that the influence of a_w_ on death rates is higher for Gram-negative bacteria than for Gram-postive bacteria. The pH of salted casings should be between 7.5 and 8, and if supplemented with phosphate, the pH increases to approximately 10 [22]. In the current study, the a_w_ was low, ranging from 0.733 in samples from China to 0.810 in samples from Mongolia, and noted pH values ranged from 7.3 in samples from Belgium and Iran to 7.8 in samples from New Zealand.

The quality of casings is also determined by contamination with anaerobic sulfide-reducing bacteria (SRB) and coagulase-positive staphylococci (CoPS), and their presence is mainly associated with sanitary conditions during processing [23,24]. Coagulase-positive staphylococci were detected in the majority of the tested samples (71.4%) at a mean level of 1.24 ± 0.63 log CFU/g. Viable CoPS were not identified only in casings produced in the UK. These bacteria were most abundant in Belgian casings, followed by Iranian and Chinese casings, and least abundant in casings from Turkey and Pakistan. In turn, SRB were detected in only 20% of the tested samples at a mean level of 0.5 ± 0.43 log CFU/g. Houben [25] also found that natural hog and sheep casings were contaminated with sulfite-reducing *Clostridium* spores whose counts did not exceed 100 CFU/g. They concluded that *Clostridium* spores are highly likely to survive the manufacturing processes of all food products. *Paraclostridium bifermentans* was the most prevalent species grown on media when contamination with anaerobic SRB was analyzed. *Paraclostridium bifermentans* is one of two described species of the genus *Paraclostridium,* ubiquitous in various mesophilic environments, including soil, marine environments, and polluted waters [26]. This bacterium has been traditionally regarded as a commensal colonizing the human gastrointestinal tract [27]. However, an increasing number of *P. bifermentans* infections have been reported in clinical settings in recent years, suggesting that this bacterium can cause various infections in humans, including brain abscess and cervical lymphadenitis [28], necrotizing endometritis [29], joint infections [30], and empyema [31]. According to Zhao et al. [32], the pathogenicity of *P. bifermentans* can be attributed to its ability to infect host cells via the Listeria Pathogenicity Island 1 (LIPI-1) genetic region. Interestingly, this region contains genes related to both the infectious life cycle and bacterial survival in the food processing environment [33]. *Staphylococcus aureus* was not detected in any of the samples, and *S. agnetis* was the most prevalent CoPS strain. *Staphylococcus agnetis* is a coagulase-variable, facultatively anaerobic, non-motile, and non-spore-forming coccus. It was initially described as a causative agent of subclinical and mild clinical mastitis in cattle [34], but recent studies have shown that *S. agnetis* is a contributing factor to lameness in broiler chickens [35]. Moreover Szafraniec et al. [35] underlined that *S. agnetis* exhibits a distinct repertoire of virulence factors found in many staphylococci. Despite the fact that *S. agnetis* is not pathogenic for humans, its occurrence in meat products may lead to the contamination of by-products and food leftovers in households, thus creating a health risk for susceptible animals. In general, in food matrices with high salt concentrations, staphylococci possess a remarkable growth advantage compared to enterococci or *Escherichia coli*, due to having a more rigid cell wall and higher internal turgor pressure [36].

*Salmonella* spp., *E.coli*, and *Listeria* spp. were not identified in any of the tested casings, regardless of their origin. These bacteria are ubiquitous in nature and are part of the normal gut flora of many animal species [37,38]. Natural casings are rarely contaminated with these pathogens, probably because they are effectively eliminated during salting. A model of inactivation kinetics given by Wijnker [22] showed that *Salmonella* and *E.coli* were relatively sensitive to the combined effect of physicochemical factors, indicating that these strains were effectively inactivated by incubation at pH 10.00, whereas *Listeria monocytogenes* and *S. aureus* had low sensitivity to pH. In another study performed by Wijnker et al. [39], the reduced survival of bacterial species in natural casings was present at different a_w_ levels. At 0.75 a_w_, the death rates were found to be approximately 1.50 log CFU/g per day for *E.coli*, 0.34 log CFU/g per day for *Salmonella*, and 0.26 log CFU/g per day for *Listeria.* Moreover, these authors underlined that these bacteria should not be positively identified after a mandatory 30-day preservation period for natural casings. Similar observations were made by Houben [25], who did not detect *Salmonella* spp. or *L. monocytogenes* in any of the 214 samples of dry-salted hog and sheep natural casings produced in China, New Zealand, and the UK. However, the culture media used in the present study support the growth of non-specific bacteria, which may have an adverse impact on human health. In the *Salmonella* testing, 11.4% of the samples were colonized by *Pseudomonas oryzihabitans* and *Pseudomonas stutzeri*. *Halomonas elongata* and *Halomonas eurihalina* were identified in 11.4% of the samples in the *Listeria* testing. The samples positive for *Pseudomonas* spp. and *Halomonas* spp. originated from Iran, Mongolia, and Belgium. *Pseudomonas* spp. are ubiquitous Gram-negative bacteria that cause various infections, particularly in immunocompromised patients, but they are also implicated in bacterial endocarditis [40]. *Halomonas* spp. have been isolated from natural salt habitats, marine animals, as well as salted foods such as traditional cheeses. These bacteria have long been recognized for their biochemical functions, such as denitrification and degradation of dangerous phenolic compounds, but recent studies have shown that *Halomonas* spp. can also cause infections in humans [41].

Heavy metal contamination of meat and other edible tissues poses a significant concern for food safety and human health due to their prolonged and irreversible bioaccumulation in human organs, especially the kidneys, liver, and spleen. Natural casings are unlikely to be contaminated with heavy metal residues because intestinal tissues do not accumulate heavy metals, as these compounds can move across the thick intestinal wall without significant binding to the tissue [42]. Therefore, the presence of heavy metals in natural casings can be attributed to the salt used in the preservation process. The salt used for processing natural casings should be food-grade and therefore should not contain any heavy metals above agreed maximum limits. However, salt may also influence the migration of heavy metals by altering the chemical environment of food [43]. This state is in accordance with study performed by Wijnker [22], showing that halophilic bacteria are introduced to casings not via the original contamination of the uncleaned animal intestines or cleaning process but via the salt used as a preservation agent. The salt used for preservation must meet the requirements included in the Codex Alimentarius but can be of different origin and produced under various conditions. The maximum permissible levels of heavy metal residues in natural casings are not regulated by EU laws; therefore, in accordance with the decision of the Chief Veterinary Officer, in the absence of legal regulations at the EU and national level, the limits given in the National Research Program for Monitoring Chemical, Biological, and Drug Residues in Food Products should be taken for interpretation of results. The plan is approved each year by the Ministry of Agriculture and Rural Development and the European Commission. The average concentrations of the tested heavy metals were low or very low, ranging from thousandths of a milligram per kg for mercury (Hg) and cadmium (Cd) to tenths of a milligram per kg for lead (Pb). The concentrations of all analyzed heavy metals did not exceed the established limits in any of the examined samples, regardless of their geographic origin, but the average concentrations of Pb were close to threshold values. Since the presented study is the first published data on heavy metal concentrations in natural casings, the results of the present study were compared with heavy metal levels in meat and foodstuffs. A global systematic study conducted by Salim et al. [44] to assess the health risks of heavy metals revealed low levels of meat contamination with arsenic and mercury, whereas lead and cadmium concentrations exceeded permissible levels in meat samples from Asia and Africa. In general, Cd and/or Pb levels appear to be significantly higher in meat, as shown by research studies conducted in Spain [45] and Iran [46], and in sausages in Pakistan [47]. Interestingly, the studies conducted by Manea et al. [48] revealed that the contents of Cd and Pb are higher in conventional products than in traditional products. When considering salt as a source of trace elements in products, Pb is generally at higher concentrations in all the analyzed samples, whereas Hg is at lower levels in the investigated samples [49]. These findings are in accordance with our results, showing Pb concentrations close to permissible upper limits and Hg levels below the LOQ in all samples. In addition, we noticed that microbial contamination was not correlated with heavy metal levels. According to Nnaji et al. [50], bacteria are highly susceptible to external factors, such as contamination with heavy metals, which can induce their alterations. The toxicity of heavy metals to various microbial species impairs cellular function by binding to protein molecules, inhibiting enzymes, or producing ROS, leading to cellular damage [51].

## 5. Conclusions

The results of this study confirmed that salting produces a final product with low microbiological contamination of natural casings, as the examined bacterial loads were below critical limits in all of the analyzed samples. However, in some cases, potentially harmful bacteria may survive food processing and can multiply during storage or handling, posing a risk to human health. The concentrations of arsenic, mercury, lead, and cadmium were within the acceptable limits; however, concentrations for Pb were close to threshold levels in several samples, which indicates that effective measures are needed to control food contamination. The study demonstrated that the quality and safety of natural casings were not affected by their geographic origin, but food safety practices by individual producers can contribute to final quality.

## Figures and Tables

**Table 1:** Microbial contamination of natural sheep casings.

Samples		Microorganisms													
Country of Origin	Number of Pooled Samples * (Mean Number of Samples in a Single Pool)	Total Counts of Aerobic Mesophilic Bacteria	*Escherichia* *coli*	*Anaerobic Sulfide-Reducing Bacteria* (SRB)		*Coagulase-Positive* *Staphylococci* (CoPS)		*Listeria monocytogenes*				*Salmonella* spp.			
		Culture Media													
		PCA	TBX Agar	ISA Agar		Baird-Parker Agar		PALCAM Agar		Oxford Agar		XLD Agar		HE Agar	
		Bacterial Growth Observed													
		Mean log_10_ CFU/g ± SD	Mean log_10_ CFU/g ± SD	Number of Positive Samples	Mean log_10_ CFU/g ± SD	Number of Positive Samples	Mean log_10_ CFU/g ± SD	Number of Positive Samples	Isolated Bacteria	Number of Positive Samples	Isolated Bacteria **	Number of Positive Samples	Isolated Bacteria **	Number of Positive Samples	Isolated Bacteria
Turkey	4 (12)	3.45 ± 2.68	0	0	ND	3	1.04 ± 0.48 ^A^	0	ND	0	ND	0	ND	0	ND
Iran	5 (18)	3.50 ± 2.84	0	2	0.97 ± 0.17 ^A^	4	1.89 ±1.17 ^B^	0	ND	2	*Halomonas* *elongata* *Halomonas* *eurihalina*	1	*Pseudomonas* *stutzeri*	0	ND
China	5 (16)	4.08 ± 3.16	0	0	ND	5	1.74 ± 1.11 ^B^	0	ND	0	ND	0	ND	0	ND
Mongolia	4 (15)	2.98 ± 2.17	0	0	ND	3	1.23 ± 0.47 ^A^	0	ND	1	*Halomonas* *elongata*	2	*Pseudomonas* *stutzeri*	0	ND
Pakistan	5 (23)	4.03 ± 3.38	0	1	0.6 ^B^	3	1.02 ± 0.3 ^A^	0	ND	0	ND	0	ND	0	ND
New Zealand	4 (18)	3.11 ± 2.14	0	2	0.81 ± 0.17 ^B^	4	1.11 ± 0.69 ^A^	0	ND	0	ND	0	ND	0	ND
United Kingdom	4 (14)	2.91 ± 2.23	0	1	0.69 ^B^	0	ND	0	ND	0	ND	0	ND	0	ND
Belgium	4 (13)	3.51 ± 2.52	0	1	0.95 ^A^	3	1.93 ± 1.17 ^B^	0	ND	1	*Halomonas* *elongata*	1	*Pseudomonas* *oryzihabitans*	0	ND

ND = not detected. * Each pooled sample refers to a separate batch (a single pooled sample consists of individual samples taken from 20% of the containers constituting the batch). ** Identified by MALDI TOF. Different letters indicate statistically significant differences (*p* ≤ 0.05).

**Table 2:** Concentrations of lead (Pb), cadmium (cd), arsenic (As), and mercury (Hg) in natural sheep casings from various countries.

Country of Origin	Number of Pooled Samples Representing Different Batches	Parameters	Pb	Cd	As	Hg
		MRL [mg/kg] *	0.10	0.05	0.20	0.01
Turkey	4	Mean ± SD [mg/kg]	0.068 ± 0.057	0.007 ± 0.01	0.028 ± 0.016	<0.001
		Range [mg/kg]	0.018–0.15	0.0012–0.022	0.011–0.049	<0.001
		n < LOQ **	0	0	0	4
		n > MRL ***	0	0	0	0
Iran	5	Mean ± SD [mg/kg]	0.068 ± 0.035	0.008 ± 0.009	0.042 ± 0.05	<0.001
		Range [mg/kg]	0.023–0.11	<0.001–0.021	<0.01–0.13	<0.001
		n < LOQ	0	1	1	5
		n > MRL	0	0	0	0
China	5	Mean ± SD [mg/kg]	0.084 ± 0.057	0.008 ± 0.009	0.054 ± 0.031	<0.001
		Range [mg/kg]	0.023–0.17	<0.001–0.018	0.14–0.094	<0.001
		n < LOQ	0	2	0	5
		n > MRL	0	0	0	0
Mongolia	4	Mean ± SD [mg/kg]	0.092 ± 0.049	0.014 ± 0.009	0.034 ± 0.027	<0.001
		Range [mg/kg]	0.045–0.16	<0.001–0.023	0.014–0.073	<0.001
		n < LOQ	0	1	0	4
		n > MRL	–0	0	0	0
Pakistan	5	Mean ± SD [mg/kg]	0.097 ± 0.052	0.01 ± 0.01	0.035 ± 0.028	<0.001
		Range [mg/kg]	0.043–0.16	<0.001–0.026	<0.01–0.083	<0.001
		n < LOQ	0	1	1	5
		n > MRL	0	0	0	0
New Zealand	4	Mean ± SD [mg/kg]	0.064 ± 0.048	0.011 ± 0.007	0.026 ± 0.014	<0.001
		Range [mg/kg]	0.017–0.13	<0.001–0.019	0.011–0.042	<0.001
		n < LOQ	0	1	0	4
		n > MRL	0	0	0	0
United Kingdom	4	Mean ± SD [mg/kg]	0.069 ± 0.045	0.008 ± 0.009	0.028 ± 0.02	<0.001
		Range [mg/kg]	0.025–0.13	<0.001–0.022	< 0.01–0.056	<0.001
		n < LOQ	0	1	1	4
		n > MRL	0	0	0	0
Belgium	4	Mean ± SD [mg/kg]	0.069 ± 0.043	0.005 ± 0.007	0.041 ± 0.036	<0.001
		Range [mg/kg]	0.033–0.13	<0.001–0.017	< 0.01–0.087	<0.001
		n < LOQ	0	1	1	4
		n > MRL	0	0	0	0
Total	35	Mean ± SD [mg/kg]	0.077 ± 0.045	0.009 ± 0.008	0.036 ± 0.029	<0.001
		Range [mg/kg]	0.017–0.17	<0.001–0.026	<0.01–0.13	<0.001
		n < LOQ	0	8	4	35
		n > MRL	0	0	0	0

* MRL—maximum residue limit for meat products according to the national program for monitoring prohibited substances and biological and chemical residues in animals and food of animal origin; ** n < LOQ—number of samples below the limit of quantification; *** n > MRL—number of samples above the maximum residue limit.

## Data Availability

The original contributions presented in this study are included in the article. Further inquiries can be directed to the corresponding author.
